# Using probabilistic linkage to improve estimates of access to services among the migrant population: The case of access to immunisation programs in Chile

**DOI:** 10.23889/ijpds.v8i2.2348

**Published:** 2023-09-14

**Authors:** Nicolas Libuy, Jorge Pacheco, Jorge Vargas

**Affiliations:** 1 Centre for Longitudinal Studies, University College London, London, United Kingdom; 2 Ministry of Health, Chile, Santiago, Chile

## Objectives

Governments often struggle to accurately estimate the number of migrants using public services due to the lack of a unique national ID. We aim to study this in the context of migrant access to immunization programs in Chile and estimate vaccine coverage in school-age children.

## Methods

To estimate vaccine coverage for migrant school-age children, we combined data from two databases: the Chilean National Immunization Register (which contained 77.9 million records) and the School Enrollment database (which contained around 68 million records, representing about 3.6 pupils per year). Using Splink, a Python package developed by the UK Ministry of Justice, we created a probability linkage model to link and deduplicate records of migrants who lack a unique national ID. The following linkage keys were considered in the model: first and second name, first and last name and date of birth. Linkage quality was evaluated using ‘gold standard data.

## Results

In 2022, we find that out of 3,644,467 students enrolled in school, 140,317 of them were migrants who didn't have a Chilean national ID. Additionally, in the NIR database, 5.2 out of 77.9 million records belonged to migrants without a national ID. After removing duplicates from both databases, our linkage model determined that 52,524 of the 140,317 students without a national ID in SE were linked to NIR (37.4%). We find that excluding migrants without national IDs when estimating national vaccine coverage for school-aged children leads to an underestimation of 2%, from 86% to 88%.

## Conclusion

Our findings emphasize the significance of utilizing linkage techniques in order to accurately estimate access to public services for migrant populations who typically lack a national ID. By linking their records across public institutions, more reliable data can be obtained.

